# Developing Biosensors in Developing Countries: South Africa as a Case Study

**DOI:** 10.3390/bios6010005

**Published:** 2016-02-02

**Authors:** Ronen Fogel, Janice Limson

**Affiliations:** Biotechnology Innovation Centre, Rhodes University, P.O. Box 94, Grahamstown 6140, South Africa; r.fogel@ru.ac.za

**Keywords:** South Africa, biosensors, nanotechnology, biotechnology, innovation, biorecognition

## Abstract

A mini-review of the reported biosensor research occurring in South Africa evidences a strong emphasis on electrochemical sensor research, guided by the opportunities this transduction platform holds for low-cost and robust sensing of numerous targets. Many of the reported publications centre on fundamental research into the signal transduction method, using model biorecognition elements, in line with international trends. Other research in this field is spread across several areas including: the application of nanotechnology; the identification and validation of biomarkers; development and testing of biorecognition agents (antibodies and aptamers) and design of electro-catalysts, most notably metallophthalocyanine. Biosensor targets commonly featured were pesticides and metals. Areas  of regional import to sub-Saharan Africa, such as HIV/AIDs and tuberculosis diagnosis, are also apparent in a review of the available literature. Irrespective of the targets, the challenge to the effective deployment of such sensors remains shaped by social and economic realities such that the requirements thereof are for low-cost and universally easy to operate devices for field settings. While it is difficult to disentangle the intertwined roles of national policy, grant funding availability and, certainly, of global trends in shaping areas of emphasis in research, most notable is the strong role that nanotechnology, and to a certain extent biotechnology, plays in research regarding biosensor construction. Stronger emphasis on collaboration between scientists in theoretical modelling, nanomaterials application and or relevant stakeholders in the specific field (e.g., food or health monitoring) and researchers in biosensor design may help evolve focused research efforts towards development and deployment of low-cost biosensors.

## 1. Introduction

The scope for biosensor research generally in southern Africa is perhaps best understood when considering the social and economic paradigms common to most developing countries and emerging economies. Table 1 offers a sample of sub-Saharan countries and summarises some of the key economic and population medical metrics, as aggregated and presented by the World Bank. In this Table, France has been selected at random as an example of the same metrics within a member state of the European Union.

Developing countries in Africa tend to combine a low-income population majority with less than optimal regulatory monitoring infrastructure. This is coupled to a heavy regulatory bias favouring the manufacturing/mining/agricultural industries as the primary means of employment. Using the *per capita* Gross Domestic Product (GDP) as a measure of economic productivity: most of the countries present in sub-Saharan Africa generate far less GDP than developed nations; accordingly, they tend to have significantly less money available for healthcare, both at public and private spending levels, as exemplified in the countries presented in Table 1. A large sector of the population is either located in remote rural areas without ready access to traditional medical care, or reside in informal peri-urban settlements with variable access to sanitation and potable water technologies. These factors, combined with the lower proportional public funding into scientific research (Table 1), are realities that drive the current research interest for on-site, cost-effective sensors capable of routine, sensitive and selective detection of a range of targeted compounds present in humans, food, water and the environment.

**Table 1:** Health and economic indicators of select sub-Saharan African countries, contrasted against France as an example of a developed European country.

Country	Per Capita GDP 2013, USD	Poverty Gap, % of Population ≤2 USD/Day/Capita (Year)	Per Capita Health Expenditure, USD (2010–2014)	R & D Expenditure, % GDP (2010–2012)
Central African Republic	333.2	n.d.	13	n.d.
Democratic Republic of the Congo	484.2	n.d.	16	n.d.
Mozambique	605.4	n.d	40	0.46
Zimbabwe	953.4	n.d.	n.d.	n.d.
Chad	1053.7	60.5% (2011)	37	n.d.
Zambia	1844.8	86.6% (2010)	93	n.d.
South Africa	6886.3	26.2% (2011)	593	0.76
France (as a comparison)	42,560.4	n.d	4864	2.25

Currency values are presented in United States dollars (USD), calculated at the dates co-presented with the values. Data aggregated and published by the World Bank [1]; n.d.—no data available.

The diffused nature of the healthcare institutions present in developing countries and the particular challenges those bring for sensor development is a feature that drives much of the approach to research. However, by the same token, many areas of Africa, and certainly South Africa, are blends of both developed and developing countries, where access to state-of-the-art health screening technologies match or better those in more developed economies. Tellingly, South Africa (Table 1), possessing the highest estimated *per capita* annual GDP of sub-Saharan countries (6886 United States Dollars, USD, as measured in 2013) and the highest total *per capita* health expenditure (593 USD), still has over a quarter of its population living on less than 2 USD *per capita* per day, highlighting the economic inequalities present in the country and the concomitant differences in access to available healthcare. This dichotomy is one that presents African scientists across the continent, and certainly in southern Africa, with a challenge to approach research such that it caters for a wider potential, global market (*i.e.*, laboratory-based technologies operated by skilled professionals) against the backdrop of the overwhelming need for rapid, accurate, low-cost sensors easily operable in remote environments that are required by a large majority of the health- and environmental-care operations on the continent.

The breadth of targets identified for biosensing, combined with the diversity of technology approaches and design considerations available means that biosensor research focus in South Africa is spread across a number of areas as evidenced in this mini-review: nanotechnology and nanoscience; identification and validation of biomarkers; development of biorecognition agents (antibodies and aptamers) and design of electro-catalysts, most notably metallophthalocyanines.

Nanotechnology-based approaches for sensor design are a common theme referred to in the areas under discussion, as is the strong focus on electrochemical sensor technology. In accordance with global trends of nanotechnology application in electroanalysis [2], and “symptomatic” of the role that nanotechnology is suggested to be able to play in developing countries [3], both themes are readily evidenced by examining biosensor research publications (Table 2). Biosensors are essentially a “biotechnology” product while biotechnology as a field of research endeavours also shapes the design and scope of sensor technologies developed in the country, in a way not dissimilar to that of the nanotechnology approach.

**Table 2:** Summary of available literature in biosensor-related fields in South Africa, categorised by analyte of interest (from 2004–2014).

Target (Biorecognition Agent)	Transducer (Transduction)	Reported LOD	Ref.	Basis of Signal Reported by Authors
***Inorganic analytes***				
AsO_3_, K_3_Fe(CN)_6_, Prussian Blue (Cytochrome c)	BDD (SWV, CV)	8.08 μM (AsO_3_)	[4]	Inhibition of cytochrome c activity, measurable as direct electron transfer from cytochrome c.
Cd^2+^ (HRP)	Maize tassel MWCNTs (Voltammetry)	>5 μg/L	[5]	Inhibition of HRP activity, measurable as electrocatalytic reduction of H_2_O_2_
Cd^2+^, Cu^2+^, Pb^2+^ (HRP)	PtE/PANI (Amperometry)	0.033 ppb (Pb^2+^)	[6]	Inhibition of HRP activity, measurable as direct electron transfer from HRP in the presence of H_2_O_2_
Cd^2+^, Pb^2+^, Hg^2+^ (HRP)	PtE/PANI-co-PDTDA (DPV)	(8−9) × 10^−4^ μg/L ~pM levels	[7]	Inhibition of HRP activity, measurable as electrocatalytic reduction of H_2_O_2_
Cu^2+^ (HRP)	GCE/Maize tassel MWCNTs (Amperometry)	~4.2 μg/L	[8]	Inhibition of HRP activity, measurable as electrocatalytic reduction of H_2_O_2_
H_2_O_2_ (HRP)	PtE/PANI nanotubes/Polyester sulphonic acid (DPV)	0.185 μM	[9]	Inhibition of HRP activity, measurable as electrocatalytic reduction of H_2_O_2_
Heavy metals and inorganic components (recombinant bacteria)	pLUX plasmid (Bioluminescence)	>20 mg/L (Pb)	[10]	Suppression of metabolic activity of transgenic *Escherichia coli* and *Shigella sonnei* bacteria, measurable as bacterial luciferase operon expression (bioluminescence)
H_2_O_2_(HRP)	Maize tassel/MWCNTs (Voltammetry)	4 μM	[11]	Inhibition of HRP activity, measurable as electrocatalytic reduction of H_2_O_2_
H_2_O_2_(HRP)	Induced nanofibril PANI/PV sulphonate polymer (Amperometry)	30 μM	[12]	Inhibition of HRP activity, measurable as electrocatalytic reduction of H_2_O_2_
Pb^2+^, Cd^2+^ (HRP)	Maize tassel MWCNTs (Voltammetry)	2.5 μg/L (Pb^2+^)	[13]	Inhibition of HRP activity, measurable as electrocatalytic reduction of H_2_O_2_
***Small organic molecule analytes***				
2,4-dichlorophenol (cytochrome P450-3A4)	GCE/Nafion/Co(SEP)^3+^	0.043 μg/L	[14]	Inhibition of cytochrome P450 activity, measurable as electrocatalytic reduction of O_2_.
Aflatoxin B_1_ (rabbit antiserum)	Pt/PANI/PSSA (EIS)	0.1 mg/L	[15]	Formation of antigen-antibody complex, measurable as increased modelled charge-transfer resistance
β-estradiol (β-estradiol aptamer)	AuE/Dendritic PPI-Polythiophene (SWV)	>0.1 nM	[16]	Formation of aptamer-target complex, measurable as decrease in the SWV current.
Broad range of organic pollutants (HRP)	PtE/PANI (Amperometry)	Qualitative	[17]	Inhibition of HRP activity, measurable as electrocatalytic reduction of H_2_O_2_
Carbamate and Organophosphate pesticides (AChE)	AuE/MBT/(poly-[o-methoxyaniline]/PDMA) /PSSA (SWV, DPV)	0.06 ppb (carbofuran)	[18]	Inhibition of AChE activity, measurable as anodic detection of acetaldehyde, produced from MBT-PDMA reduction of acetate, produced during AChE reaction with acetylcholine
Catechin (apple polyphenol oxidase)	Carbon paste, 20% w/v green apple/GCE (DPV)	1.76 ppb	[19]	Production of enzyme-catalysed oxidation products, measurable as electroactive compounds
Chemical Oxygen Demand (*Shigella* spp.)	pLUX plasmid (Bioluminescence)	n.r.	[20]	Wastewater strength measured by increase in metabolic activity of transgenic *Shigella* bacteria, as described for Ref. [10], above
Diazinon (HRP)	PtE/PANI/ASA (Voltammetry)		[21]	Inhibition of HRP activity, measurable as electrocatalytic reduction of H_2_O_2_
Glyphosate (HRP)	AuE/PDMA/PSS	1.70 μg/L	[22]	Inhibition of HRP activity, measurable as electrocatalytic reduction of H_2_O_2_
Glyphosate and aminomethylphosphonic acid (HRP)	AuE/PDMA/PSS (Amperometry)	0.16 μg/L and 1 μg/L, respectively	[23]	Inhibition of HRP activity, measurable as electrocatalytic reduction of H_2_O_2_
Indinavir (Cytochrome P450-3A4)	PtE/didodecyldimethylammonium bromide vesicle/BSA (Amperometry)	61.5 μg/L	[24]	Inhibition of cytochrome activity, measurable as direct electron transfer from cytochromes in presence of O_2_.
l-Tyrosine (Tyrosinase)	BDD, PANI entrapped (SWV)		[25]	Electrocatalytic oxidation of l-tyrosine in the presence of tyrosinase.
Organophosphate pesticides (AChE)	Au/MBT/PANI/AChE/PVAc (Voltammetry)	0.018 nM (Chlorpyrifos)	[26]	Inhibition of AChE activity, as described for Ref [18] above
Organophosphates (AChE)	AuE/MBT/PANI/AChE/PVAc (amperometry)	0.147 ppb (Diazinon)	[27]	Inhibition of AChE activity, as described for Ref [18] above
Phenolic compounds (Laccase)	GCE/BSA and glutaraldehyde (Amperometry)	~μM range	[28]	Production of enzyme-catalysed oxidation products, measurable as electroactive compounds
Phenolic compounds (Laccase)	GCE/Graphite paste (DPV)	n.r.	[29]	Production of enzyme-catalysed oxidation products, measurable as electroactive compounds
Rifampicin (cytochrome P450-2E1)	AuE/PVP-AgNPs/poly(8-anilino-1-naphthalene sulphonic acid (DPV)	~50 nM	[30]	Electro-reduction of the cytochrome-rifampicin complex, driving catalysis
Urea (Urease)	ZrO_2_ NPs-PPI (Amperometry)	>0.01 mM	[31]	Detection of urease-catalysed production of NH_3_, detectable by anodic detection of NH_3._
***Biopolymer analytes***				
(+)-3,3′,5-Triiodo-L-thyronine(Antiserum)	Carbon paste (amperometry)	2.19 ng/mL	[32]	Not reported on
Anti-Mycolic acid IgG (Mycolic acids)	IAsys affinity biosensor (Refractive indices)	Qualitative	[33]	Binding of host IgG to attached mycolic acids, measured as changes in refractive indices of films on sensor cuvettes
Antitransglutaminase antibody (Transglutaminase antigen)	GCE/Overoxidised polypyrrole/Au NPs (EIS)	>1 μM	[34]	Formation of antigen-antibody complex, measured as increase in modelled charge-transfer resistance.
β-d-glucuronidase activity (*Moraxella* sp. bacteria)	GCE	2 CFU/100 mL	[35]	Anodic detection of more sensitive microbial metabolite from enzyme-catalysed product of p-nitrophenyl-β-d-glucuronide
Creatine and Creatinine (creatinase, creatininase sarcosine oxidase)	Monocrystalline Diamond Paste (Amperometry)	1 × 10^−3^ fM	[36]	Amperometric detection of enzyme-catalysed generation of H_2_O_2_ from creatine degradation; conversion of creatinine to creatine.
Entantiomers of enalapril, ramipril and pentopril (l-amino acid ) oxidase	Carbon paste (Amperometry)		[37]	Not reported
Ethambutol (cytochrome P450-E21)	AuE/poly (8-anilino-1-napthalene sulphonic acid)/Ag NPs (Amperometry, voltammetry)	0.7 μM	[38]	Electro-reduction of the cytochrome-ethambutol complex, driving further catalysis, measurable as the reduction of Fe^3+^ centre of the cytochrome
Fluoxetine (Cytochrome P450)	GCE/PANI (Amperometry)	~1 nM	[39]	Cathodic detection of complex-catalysed product of Fluoxetine.
Glucose (Glucose oxidase)	PPI dendrimer/GCE (Amperometry)	0.1 mM	[40]	Anodic detection of enzyme-generated H_2_O_2_ in presence of substrate
Glucose (Glucose oxidase)	GCE/Co(II)phthalocyanine-cobalt(II) tetraphenylporphyrin pentamer complex (Amperometry)	10 μM	[41]	Anodic detection of enzyme-generated H_2_O_2_ in presence of substrate
Glucose (Glucose oxidase)	AuE/β-mercaptoethanol/ (Amperometry)	0.4 μM	[42]	Anodic detection of enzyme-generated H_2_O_2_ in presence of substrate
gp120 protein (biotinylated gp120 aptamer)	GCE/dendritic PPI/streptavidin (EIS)	0.2 nM	[43]	Formation of aptamer-target complex, measured by increased modelled charge-transfer resistance
Immunoglobulins (Lysozyme)	3-mercaptopropionate succinimide/ZnO nanowires (Potentiometry)	103 ng/mL	[44]	Formation of antigen-antibody complex causes bending of or applies tensile pressure to nanowires, measurable as change in piezoelectric potential.
Measles antigen (HRP-linked IgG)	AuE/phenylethylamine/ glutaraldehyde/antigen/BSA (Voltammetry)		[45]	Binding of HRP-linked secondary antibody to primary antibody-antigen complex; Electrochemical detection of HRP-catalysed oxidation products of TMB.
Single-stranded DNA (complementary DNA)	GCE (Voltammetry, EIS)	<5 nM	[46]	Hybridisation of DNA molecules, measurable as a decrease in modelled charge-transfer resistance.
Single-stranded DNA (complementary DNA)	ITO/Chitosan-AuNP-mercaptopropionate (Voltammetric detection of Fe(CN)_6_^3−/4−^)	0.03 fM	[47]	Hybridisation of DNA molecules, measurable as an increase in Fe(CN)_6_^3−/4−^ current reasponse.
Single-stranded DNA (complementary DNA)	AuE/Co(II) salicylaldiimine metallodendrimer (EIS)	0.34 pM	[48]	Hybridisation of DNA molecules, measurable as an increase in modelled charge-transfer resistance
Single-stranded DNA (complementary DNA)	GCE/PPI/AuNPs (EIS)	~pM levels	[49]	Hybridisation of DNA molecules, measurable as an increase in modelled charge-transfer resistance

Abbreviations and contractions: AChE—Acetylcholinesterase; AuE—Gold electrode; BDD—Boron-doped diamond electrode; BSA—Bovine Serum Albumin; CV—Cyclic Voltammetry; DPV—Differential Pulse Voltammetry; EIS—Electrochemical Impedance Spectroscopy; GCE—Glassy carbon electrode; HRP—Horseradish peroxidase; IgG—Immunoglobulin G; ITO—Indium Tin Oxide Electrode; MBT—mercaptobenzothiazole; MWCNTs—Multi-walled carbon nanotubes; NPs—Nanoparticles; PANI—polyaniline; PDMA—poly(2,5-dimethoxyaniline); PDTDA—poly(2,2’-dithiodianiline); PPI—poly (propylene imine); PSS—poly(4-styrenesulfonic) acid; PtE—Platinum electrode; SWV—Square Wave Voltammetry.

While not an exhaustive reporting of the available literature, this review serves to identify key research interests, as well as some interesting features of the research landscape currently emerging from South Africa in the development of biosensors. This review also explores in brief the positioning of the research in biosensor development within the context of national policy (most notably biotechnology and nanotechnology), the country’s infrastructure and resources.

## 2. Transducer Types and Sensor Fabrication Materials

Examination of the biosensor transducer types in Table 2 [4,5,6,7,8,9,10,11,12,13,14,15,16,17,18,19,20,21,22,23,24,25,26,27,28,29,30,31,32,33,34,35,36,37,38,39,40,41,42,43,44,45,46,47,48,49] reveals an overwhelming amount of research originating from South Africa has been focused on electrochemical (amperometric, impedimetric, potentiometric and voltammetric) configurations of biosensors, with only a few publications found investigating colourimetric/photometric bases for signal generation. This is, itself, due to the many commonly reported beneficial properties of electroanalysis over other transducers—e.g., low fabrication-cost, consistency of fabrication, high degree of alloweable automation, ability to derive multiple signals from a single analytical device (signal multiplexing), lower analytical equipment cost requirements, *etc*.

This bias in favour of electroanalytical sensor technology is not unexpected and may be influenced by the promise thereof as a rapid and low-cost technology for qualitative testing in remote areas, given the lack of access to diagnostics in remote areas.

A wide variety of materials, beyond the functional biorecognition agents that underpin biosensor technologies, have been incorporated into biosensors researched in South Africa (Table 2). Nanomaterials and metallophthalocyanines, in particular, are identified as being of apparent interest within the community of biosensor researchers identified during the compilation of this mini-review and are discussed in forthcoming sections. In addition, the interaction between nanomaterials, biorecognition agents and functional polymers are also intensively researched in South Africa, and are briefly discussed below.

### 2.1. Nanomaterials in Reviewed Biosensors

In recent years, nanotechnology has been repeatedly touted as a technological route potentially capable of solving many of the current global issues. In a recent review of the future application of nanotechnology within developing countries within the sectors of water treatment, human health and agriculture (arguably the most fundamental societal parameters required for good governance and economic development), nanomaterial biosensors (largely within the agriculture sector) were specifically identified as desirable future products [3]. This keen research interest is borne out in the number of nanoscale applications approved for publication over the last decade, with the developing nations contributing a significant portion of research within the area. The specific promise of nanotechnology in the realm of biosensors has long been anticipated.

For the above reasons, the rational design and fabrication of nanoscale structures has been under intense investigation for application in sensors. In South Africa, these are mainly focused on the application of metal nanoparticles, largely gold, and carbon-based nanomaterials (notably carbon nanotubes). These are applied as directly to functionalise surfaces, or as composites with other materials (Table 2) and frequently employed within electrochemical sensor designs.

Nanomaterial use and applications are intimately associated with electroanalytical methods of sensor research in the country. The convergence of nanomaterials and electrochemical sensors in the biosensor field (Table 2) is a reflection of a wider global phenomenon in biosensor research towards the development of so-called nanocomposite biosensors [2]. South African research utilising nanomaterials is further supported by manufacturing of platinum group metal nanoparticles, thanks largely to the country’s mineral wealth and associated research in its minerals technology parastatal, Mintek.

Specifically, electrochemical nanocomposite electrodes offer further advantages over conventional fabrication techniques: through improving effective electrode surface area via the use of electro-conductive nanostructures; as catalysts within their own right and enhancing control of the local microenvironment [2]. In the instance of noble metals, the high surface area to volume ratio provided through the inclusion of nanostructures improves the affordability of the overall electrode, rather than constructing the macroelectrode from the noble metal itself [2].

It is well-established that, in order to improve upon sensor response, an adequate dispersal of the catalyst(s) at/near the transducer is essential [2]. An additional associated area of research, in the formation of nanocomposite materials ensuring the adequate dispersal of the nanoparticles within the composite materials, is also emerging. The topical nanomaterial, graphene serves as an excellent example of the current research debate regarding nanostructure dispersal. The electronic, physical and chemical properties of graphene have been established to be largely influenced by the degree of dispersion, significantly influencing the electrochemical functionality of this nanomaterial [50]. Aggregation of the nanoparticles typically results in lowered catalytic efficiencies and, critically for the purposes of electrochemical biosensors, lowered conductivity and increased impedance [50]. This has given rise to an interesting and ongoing research enquiry to ensure adequate dispersal of nanostructures [50] (and nanostructure/biomolecule composite materials) currently occupies a great deal of current nanochemistry and nanosensor literature. The use of additional modifications intended to disperse and support nanomaterials to optimise their intended functions has been researched, both globally and in South Africa and are discussed in their relevant sections, below.

A wide variety of metallic nanoparticles [31,38,44,47,49], carbon nanotubes [5,8,11,13], and polymer-derived nanostructures [9,12,23] have been included during fabrication of electrochemical biosensors by various South African groups. The various benefits reported by the inclusion of the nanomaterials documented in these publications are similar to those described above.

### 2.2. Metallophthalocyanines—Widely-Researched Chemical Catalysts in Reviewed Publications

The catalytic properties of metallophthalocyanine (MPc) species are strongly represented as chemosensor agents in their own right by several research groups across South Africa, especially with regard to adapting their rich reduction/oxidation chemistry for electrochemical sensor purposes [51]. Research directions of this class of molecules within sensors are manifold: the design and synthesis of novel MPcs; their application as electrocatalysts for signal generation/amplification in the detection of numerous analytes; and in bio- and nanocomposite assemblies where they may play supportive roles as dispersal agents for nanomaterials and utilising nanomaterials for immobilisation support. Several excellent reviews on the topic of MPc in electrochemistry exist, noting the increased trend towards the utilisation thereof in sensor development [51]. A further noteworthy review [52] collectively studies the application and utility of MPcs in hybrid assemblies along with nanomaterials for sensing purposes.

Metallophthalocyanines included in composite assemblies (with enzymes) [41,42] for biosensor applications, are described as serving to enhance catalytic detection of the target analyte and also as a support for the biorecognition agent through covalent coupling [53].

Interestingly no publications originating from South African groups were found utilising the combined performance enhancing characteristics of MPc-nanomaterial composites in conjunction with enzymes, antibodies, whole cells or aptamers for biosensing applications, possibly presenting a research opportunity for those groups engaged in similar research.

### 2.3. Polymeric Supports in Reviewed Publications

Polymeric supports have also found a unique role in nano- and biosensor fabrication in South Africa, both as dispersants for functional elements of biosensors, and as functional elements in their own rights. A notable example is work cited here on aniline-derived polymers for electrochemical biosensor fabrication. The inclusion of polyaniline (PANI) as a conductive polymer in electrochemical sensors is well-established and its continued use, both as a polymer [15,25] and a co-polymer [7], is a testament to its stability and ease of preparation and tunable chemical and electrochemical properties that have made it a mainstay in many reported sensor configurations [54,55] both globally and within South African research. Work at the University of the Western Cape, highlights the flexible methods of preparation of polyaniline as a conductive immobilisation surface for redox-active enzymes as both sole polymer [6,17,39] and co-polymer [7,25], while the tunability of the polymer’s properties has been iteratively investigated using biosensors constructed by (amongst others): sulphonated dopants to the preparation of PANI, [9] and sulphonated chemical derivatives of PANI e.g., poly(2,5-dimethoxyaniline), PDMA (Table 2) [18,19,20,21,22,23], which has culminated in recent years to the characterisation and performance analysis of polyaniline-based nanostructures [9,12,21] fabricated directly onto electrode surfaces. As both a model enzyme system and as a biorecognition agent for the detection of H_2_O_2_ and a wide variety of inhibitory compounds, e.g., metals, the enzyme horseradish peroxidase (HRP) has been used extensively in the design and fabrication of these electrochemical sensors (Table 2) [5,6,7,8,9,11,12,17,21,22,23] . Similar inhibitory bases for biosensor signals have been investigated by this group using acetylcholinesterase [18] and tyrosinase [25] enzymes and cytochrome enzyme complexes [39].

Additional research examined the use of polymers to aid in the dispersion of nanomaterials, e.g., ZrO_2_ nanosphere catalysts [31], gold [49] and silver [38] nanoparticles and as a means of efficiently dispersing and attaching enzymes to transducer surfaces [31,38,40].

### 2.4. Biomolecular Supports for Biosensor Materials

Inert proteins, such as bovine serum albumin [28,45] have been investigated as a means for improving the stability of protein-based sensors. The dispersal of nanomaterials using biologically-derived biopolymers (chitosan) is also represented in the reviewed literature [47], as has become common in recent years [56]. As an interesting variation of this research area, maize tassel—the thin, cellulose-rich, fibrous material located on the inner husk of maize (*Zea mays*) cobs—has recently been demonstrated as a novel and inexpensive method of dispersing carbon nanotubes [5,8,11,13]. Physisorption of carbon nanotubes onto compacted maize tassel has been experimentally demonstrated, and the resulting composite used for the immobilisation of biomolecules for biosensor purposes, highlighting some of the best aspects of nanomaterial support—the attachment of carbon nanotubes to the maize tassel confers upon the maize tassel electrical conductivity, allowing novel electrode materials to be fabricated with this [5,13]. The diverse functional groups on the maize tassel are thought to provide a gentler method of enzyme immobilisation, providing superior attachment to the solid support.

## 3. Biorecognition Agents Reviewed in Publications

The search for new technologies that can advance the biomedical field, particularly the early detection of disease, is a well-known driver for the development of biorecognition agents for biosensor assembly [57]. As such, South African researchers are following global trends in the investigation of both immunosensor fabrication and operation to the more recent aptamer and aptasensor technologies emerging across the world.

### 3.1. Immunosensors

While the immobilisation of suitable enzymes and catalysts are the traditional manner of producing electrochemical biosensors, the use of immunomolecules has also been improved upon. Researchers have demonstrated that the electrochemical detection of the common ELISA chromophore, 3,3′,5,5′-tetramethylbenzidine (TMB), can be used to create an electrochemical biosensor detecting the measles Antigen Protein via HRP-labelled secondary antibodies, in a similar configuration to what already occurs in conventional (colourimetric) ELISA systems [45]. The amperometric detection of the human thyroid hormone (+)-3,3′,5-Triiodo-L-thyronine (L-T_3_), and discrimination against the pro-hormone L-T_4_, was improved through the selective retention at carbon paste electrodes using anti-L-T_3_ antisera [32]. Similarly, inclusion of biological antigens as biorecognition agents of sensors allowed for the diagnosis of gluten intolerance [34], through the detection of antibodies produced to resist these disease states.

### 3.2. Aptamer Based Sensors

A strongly emergent thrust in South African sensor research is in the area of aptamers with researchers partnering with molecular biologists in the generation of new novel aptamers as part of an overall thrust in incorporating these into sensor assemblies [58]. Most noteworthy has been the focus of such research where targets are largely focused on disease states of relevance to sub-Saharan Africa in areas of HIV, TB and malaria.

Aptamers are single-stranded olignoucleotides capable of selective and stringent binding to a range of compounds, generated during well-established sensor processes. In addition to the ease of synthesis, the selection of nucleic acids as biorecognition agents in biosensors is noted to have several benefits in terms of storage, e.g., Table 2, [47]. Promising aptamers targeting tuberculosis [59]; HIV [60]; CD4 and malarial lactate dehydrogenase have been published and/or patented over the past decade engaging South African research groups. Translating this work into biosensors utilising aptamers evidences collaboration with groups largely in electrochemical biosensor research, with current research including a 17β-estradiol aptamer biosensor [16] for endocrine disrupting compound detection; a gp120 aptamer biosensor for HIV detection [43]. The primary methods of signal transduction are either impedimetric measurement using ferri-/ferrocyanide redox probes [16,43] or voltammetric detection of the same [16]. Both of these methods rely on the inherent electrostatic repulsion occurring between the redox probes and the negatively-charged nucleic acid aptamers to track changes in the conformation and/or complexation state of the bound aptamers; this method of monitoring biorecognition extends from genosensor work performed in South Africa [46,47,48,49]. Fluorimetric biosensors using aptamers selective for underglycosylated mucin-1 protein in breast cancer detection has also been reported [61].

## 4. Targets in Biosensor Development

An examination of Table 2 shows a wide range of targets examined, following traditional areas in sensor development, covering environmental, food and human health. Table 2 divides these into small molecules/metal ions, biopolymers, organic compounds/metabolites and whole organisms. We highlight features of interest in each of these groups.

### 4.1. Metals

A study published in 2008 ([62]) demonstrated the feasibility of maize tassel as a biosorbent, initially applied to the rapid removal of aqueous metal ions, e.g., lead, followed by several papers examining this functionality towards other metals including cadmium and copper [63]. The use of maize tassel features in several publications [5,8,13] for enhanced detection of metals where the inhibition of horseradish peroxidase was used as the detection principle. The inhibition of horseradish peroxidase for metal detection was utilised in other studies [6,7] for metal detection.

### 4.2. Biopolymers and Organisms

Of the biopolymer biosensors listed in Table 2, those for targeting disease were largely of relevance to developing countries. The detection of aflatoxin B1, in an immunosensor configuration [15], a fungal mycotoxin produced, is a concern largely in rural areas where incorrectly stored maize contaminated with fungi produce these carcinogenic secondary metabolites with serious health implications. Aflatoxin B1 has been implicated in liver disease and is of particular concern in areas of high HIV infection given its immunosuppression properties.

Tuberculosis diagnosis via detection of circulating antibodies generated by the host against the mycolic acids produced by the tuberculosis *Mycobacterium* [33] was an important breakthrough for future rapid tests in the country, but certainly worldwide, given the emergence of multi-drug resistant tuberculosis. Biosensor assemblies for detection of drugs used to treat tuberculosis [30,38] and HIV/AIDS [24] utilising cytochrome p450 assemblies has particular relevance as part of treatment monitoring strategies. Aptamer targeting of tuberculosis markers will certainly support in time efforts in this regard.

The rapid detection of coliforms as an indicator of foecal contamination is hampered by long turnaround times, limiting rapid response to water contamination. Electrochemical biosensor configurations based on the detection of enzymes of coliform origin, *B*-d-glucuronidase for *Escherichia coli* detection [35], aimed to reduce standard detection times from around 48 h to a matter of minutes. Given developing world concerns and limitations in provision of potable water, rapid monitoring of the coliform bactera in water holds substantial public health benefits, in particular for children.

Sensor assemblies utilising ZnO nanowires [44] as an indicator of infection with pathogenic microorganisms, via detection of immunoglobulins, is an example of piezoelectric transduction technologies under examination and takes a fundamental look at biosensor design and construction. Supporting such studies is work aimed at improving understanding of the state of enzymes in biosensor assemblies once immobilised. Such fundamental studies [64,65] using piezoelectic measurements at a quartz crystal microbalance support and evidence efforts at development of biosensors for commercial applications. As per international trends, South African researchers also utilise the glucose sensor as a benchmark for testing new sensor configurations and designs [40,41,42], in addition to HRP [6,7,8,9].

### 4.3. Organic Compounds/Small-Molecule Metabolites

Environmental electrochemical biosensors for pesticides (in particular of organophosphates [18]) and herbicides are a strong theme for biosensor publications from South Africa, and indeed a common application for biosensor targets amongst literature from the BRICS countries (Brazil, Russia, India, China and South Africa) [2]. Biosensors for organophosphates utilizing the inhibition of acetylcholinesterase are reported at gold composite polyaniline sensors showing good solvent compatibility and nanomolar detection limits for malathion and chlorpyrifos [26] and for diazinon [27].

Research on detection of phenolic substrates has been applied for total polyphenol content determination in wine at glassy carbon electrodes (GCE) modified with apple paste (as a source of polyphenol oxidase) [19] and at laccase modified GCE for phenolic content in an antioxidant assay of herbal tea [29]. Addressing one of the fundamental issues in phenolic based sensors using laccase enzymes, [28] describes a method for predicting the suitability of amperometric laccase sensors for different phenols.

## 5. Policies, Facilities and National Opportunities

Given the current and future prospects of nanotechnology in the field of biosensors, it is important to discuss the developing interest in nanotechnology occurring in South Africa. Currently, members of the BRICS countries (Brazil, Russia, India, China and South Africa) publish more nanotechnology-related journal articles than any other countries within their Human Development Index Group [3].

A leading aspect of South Africa’s growth in nanotechnology research is intimately associated with its National Nanotechnology Strategy of 2005, which has funded research, collaborative networking events (certainly between the BRICS, in particular between South Africa, Brazil and India), scientific meetings and, crucially, equipment. Access to state-of-the-art nanotechnology equipment through the Department of Science and Technology-led National Nanotechnology Equipment programme has been one of the key aims of the South African government in addressing equipment deficits in the country, as a means of growing nanotechnology research and development at a national level [66]. National equipment facilities can be found at most universities in the country with the most notable facilities housing key collections of nanotechnology-based equipment (under one roof) being at Rhodes University’s Centre for Nanotechnology Innovation, Nelson Mandela Metropolitan University’s Centre for High Resolution Transmission Electron Microscopy and at the National Centre for Nano-structured Materials of the Council for Scientific and Industrial Research.

This focus on nanotechnology funding and the provision of this budget led to the formation of Nanotechnology Innovation Centres (2007) at four sites within the country, each focusing on different aspects of the applied areas of the field [66]. A specific research centre was formed focusing on sensors formally housed within Rhodes University’s Centre for Nanotechnology Innovation. The utilisation of nanomaterials in sensor development is a common feature at all universities engaged in primarily electrochemical biosensor development as evidenced in Table 2 in publications emanating from institutions including University of the Western Cape (housing a dedicated Sensorlab), University of the Witwatersrand, University of Pretoria, University of KwaZulu Natal, University of Johannesburg, Rhodes University, Durban University of Technology and University of South Africa.

The national drive for increasing collaborative access to equipment and facilities also supports, in part, the country’s drive to innovate and develop products, as evidenced by several nationally approved strategies and development plans, most notably the country’s Department of Science and Technology Ten Year Plan for South Africa (2008–2018); (titled: Innovation towards a knowledge-based economy) and its National Research and Development Strategy. Within several of the national strategies, sensor and diagnostics are listed as either key technologies or enablers of other technology developments.

In a more directed manner, national funding of the Nanotechnology Innovation Centre in Sensors, has supported a development node based in Mintek, South Africa’s national mineral research organisation, which seeks to further develop research from its higher education institutional partners into commercial products. In this regard, recent successes from Mintek include its prototypes for detection of measles [45] and emerging work for targeting neglected diseases. Mintek also develops simple lateral flow diagnostics. Linked as biosensors are to the field of biotechnology, there is often an overlap in the aims and outcomes of different strategies, in a manner that supports the overall landscape synergistically. A recently launched National Bioeconomy Strategy (2014) builds on the original National Biotechnology Strategy of 2001 in that it seeks to accelerate product and prototype development, identifying enablers to improve on the poor track record of translating its bio-based research into viable commercialisable solutions.

This legislative emphasis on supporting biotechnology research has, more generally, evolved research capacity and products in cognate disciplines for biosensor development such as: biomarker discovery, (for example in tuberculosis diagnostics [57,67]); proteomics and its role in biomarker discovery [68] and aptamer generation (as detailed in this document).

## 6. Conclusions

Biosensor research in South Africa follows international trends in terms of its application of electrochemistry and nanotechnology. Many papers are aimed at new biosensor design approaches (new materials, catalysts, immobilization approaches, for example) or are aimed at conducting fundamental studies in sensor design. In terms of targets these also are not dissimilar to that studied internationally. Pesticides feature strongly (as has been noted elsewhere [3]), as do metals, as biosensor targets. There are relatively fewer publications for biosensors with targets such as TB and HIV published. Of the literature reviewed in this article, few publications appear to report on developing and applying biosensors to real-world matrices for health [32,33], food [19], or environmental [17,35] biosensing. Mostly, publications appear to showcase the utility of the designed sensor, rather than solely aiming to substantially advance the detection of the reported target. This is not an uncommon approach within the literature: complementary nucleic acids, horseradish peroxidase, acetylcholinesterase and glucose oxidase are common model biorecognition agents used globally for testing other aspects of a biosensor’s construction, and all feature prominently as such in Table 2. If usable sensor technology—especially, low-cost technology—is to be advanced, collaboration between scientists in biosensor design and experts within the relevant target sectors such as food or health may help drive focused sensor technology. While biosensor design and construction remains an important consideration for the future success of commercially-relevant sensors, demonstration of the technology performance in the specified matrices for defined targets (working with relevant stakeholders in the specific field) is a key step in the path towards commercial biosensors.

Indeed, the biosensor field may benefit from collaborative approaches in several areas including the development of new materials for biosensor assemblies. Substantial scope exists in this area through collaborations with scientists in nanomaterials research. Theoretical modeling of biosensor assemblies represents an additional area.

Substantial scope exists for research (and associated investment) that addresses the need for point-of-care diagnostics in the healthcare setting. The Ebola outbreak of 2014 reminds us again of the need to focus both on low-cost diagnostics of relevance to the developing world, but of the need to accelerate technology transfer of such research.

Despite several national strategies aimed at providing support for research commercialisation and innovation and despite the investment in fundamental research there is not yet a biosensor industry capable of commercialising biosensors for widespread use in the country. This lack of commercialisation or technology transfer is not solely a concern within the biosensor sector and may be explained by the poor links existent between research performed in higher education institutes (accounting for the greatest proportion of research papers in biosensors) and the industrial sector [3]. However, introduction of technology transfer officers at most tertiary institutions and the increasing access to basic business and entrepreneurship training for postgraduate researchers, a growing pool of skilled, entrepreneurially-minded graduates may pave the way for bridging the gap between laboratory-based biosensors and a commercial market. To unlock these opportunities, greater government investment will be needed to support technology transfer from higher education institutions. Ideally such interventions need to be focused on developing innovation spaces geographically and organisationally close to higher education.

Basic lateral flow diagnostics are however being produced commercially within the country and represent a key opportunity for developing low-cost rapid diagnostics that could in part support the great demand for point-of-care diagnostics in the healthcare sector. Certainly, opportunities exist to expand, for example, research in developing biorecognition agents (such as aptamers) which can be utilised in a range of biosensor assemblies and indeed in lateral flow diagnostics.
